# Supplementary material for: Poria cocos Polysaccharide Ameliorated Antibiotic-Associated Diarrhea in Mice via Regulating the Homeostasis of the Gut Microbiota and Intestinal Mucosal Barrier
Source: Int J Mol Sci. 2023 Jan 11;24(2):1423. doi: 10.3390/ijms24021423 (PMC9862632; doi:10.3390/ijms24021423)
Supplement: Supplementary file 1 [file ijms-24-01423-s001.zip › ijms-2061360-SI.pdf]

Supplemental Methods and Materials

Poria cocos polysaccharide ameliorated Antibiotic-Associated Diarrhea in Mice via regulating the homeostasis of the gut microbiota and intestinal mucosal barrier

Huachong Xu[1,2,†], Shiqi Wang [1,†], Yawen Jiang[1], Jialin Wu[1], Lili Chen[1], Yujia Ding[1], Yingtong Zhou[1], Li Deng[1,2,*] and Xiaoyin Chen[1,2,*]

1. immunofluorescence staining

1.1 Deparaffinize and rehydrate: incubate sections in two changes of xylene, 15 min each. Dehydrate in two changes of pure ethanol for five min, followed by dehydrate in gradient ethanol of 85% and 75% ethanol, respectively, five min each. Wash in distilled water.

1.2 Antigen repair: tissue section filled in citric acid antigen repair buffer (PH6.0) repair box in a pressure cooker with a certain amount of water, induction cooker heating to vent to spray, stop heating, release pressure, put the slice in the repair box, induction cooker heated to vent to spray, five min after close the induction cooker, the process should prevent the buffer excessive evaporation, do not dry sheet. After natural cooling, the glass slides were placed in PBS (PH7.4) and washed by shaking for three times for five min each time.

1.3 Circle and Serum blocking: eliminate obvious liquid, mark the objective tissue with liquid blocker pen. Add 3% BSA to cover the marked tissue to block non-specific binding for 30 min. Cover objective area with 10% donkey serum (for the case of primary antibody originated from goat) or 3% BSA (for the case of primary antibody originated from others)

1.4 Primary antibody: throw away the blocking solution slightly. Incubate slides with primary antibody (diluted with PBS appropriately) overnight at 4 ℃, placed in a wet box containing a little water.

1.5 Secondary antibody: wash slides three times with PBS (pH 7.4) in a Rocker device, five min each. Then throw away liquid slightly. Cover objective tissue with secondary antibody (appropriately respond to primary antibody in species), incubate at room temperature for 50 min in dark condition.

1.6 DAPI counterstain in nucleus: wash three times with PBS (pH 7.4) in a Rocker device, five min each. Then incubate with DAPI solution at room temperature for ten min, kept in dark place.

1.7 Spontaneous fluorescence quenching: wash three times with PBS (pH 7.4) in a Rocker device, five min each. Add spontaneous fluorescence quenching reagent to incubate for five min. Wash in running tap water for ten min.

1.8 Mount: Throw away liquid slightly, then coverslip with anti-fade mounting medium.

1.9 Microscopy detection and collect images by Fluorescent Microscopy. DAPI glows blue by UV excitation wavelength 330-380 nm and emission wavelength 420 nm; FITC glows green by excitation wavelength 465-495 nm and emission wavelength 515-555 nm; CY3 glows red by excitation wavelength 510-560 nm and emission wavelength 590 nm.

2. 16S rDNA for cecal microbiota

2.1 Library Construction

Variable regions V4 of bacterial 16S rRNA gene was amplified with degenerate PCR primers, 515F (5'-GTGCCAGCMGCCGCGGTAA-3') and 806R (5'-GGACTACHVGGGTWTCTAAT-3'). Both forward and reverse primers were tagged with Illumina adapter, pad and linker sequences. PCR enrichment was performed in a 50 μL reaction containing 30ng template, fusion PCR primer and PCR master mix. PCR cycling conditions were as follows: 95°C for three minutes, 30 cycles of 95°C for 45 seconds, 56°C for 45 seconds, 72°C for 45 seconds and final extension for 10 minutes at 72°C for 10 minutes. The PCR products were purified using Agencourt AMPure XP beads and eluted in Elution buffer. Libraries were qualified by the Agilent Technologies 2100 bioanalyzer. The validated libraries were used for sequencing on Illumina HiSeq 2500 platform (BGI, Shenzhen, China) following the standard pipelines of Illumina, and generating 2 × 250 bp paired-end reads.

2.2 Sequencing and bioinformatics analysis

Raw reads were filtered to remove adaptors and low-quality and ambiguous bases, and then paired-end reads were added to tags by the Fast Length Adjustment of Short reads program (FLASH, v1.2.11) [49] to get the tags. The tags were clustered into OTUs with a cutoff value of 97% using UPARSE software (v7 .0.1090) [50] and chimera sequences were compared with the Gold database using UCHIME (v4.2.40) [51] to detect. Then, OTU representative sequences were taxonomically classified using Ribosomal Database Project (RDP) Classifier v.2.2 with a minimum confidence threshold of 0.6, and trained on the Greengenes database v201305 by QIIME v1.8.0[52]. The USEARCH_global [53] was used to compare all Tags back to OTU to get the OTU abundance statistics table of each sample.Alpha and beta diversity were estimated by MOTHUR (v1.31.2) [54] and QIIME (v1.8.0)[52] at the OTU level, respectively. Sample cluster was conducted by QIIME (v1.8.0) [52] based on UPGMA. KEGG and COG functions were predicted using the PICRUSt software [55]. Barplot and heatmap of different classification levels was plotted with R package v3.4.1 and R package "gplots", respectively. The Venn plots in OTUs or in taxa were plotted with R package "VennDiagram" version 3.1.1. Species accumulation curves was plotted with R package version 3.1.1. Principal component analysis (PCA) in OTUs was plotted with R package "ade4". Partial least-squares discrimination analysis (PLS-DA) was performed by

R package mixOmics. OTU Rank curve was plotted with R package version 3.1.1. Phylogenetic tree of species was performed using FastTree (v2.1.3). Principal Coordinate Analysis (PCoA) was performed by QIIME (v1.8.0) [52] UPGMA cluster and abundance map was performed by phytools and R package version 3.5.1. LEfSe cluster or LDA analysis was conducted by LEfSe. Significant Species or function were determined by R (v3.4.1) based on Wilcox-test or Kruskal-Test. Species network analysis was performed using R (v3.4.1) and Cytoscape.
